# Management of Intestinal Strictures Post Conservative Treatment of Necrotizing Enterocolitis: The Long Term Outcome

**DOI:** 10.21699/jns.v5i3.379

**Published:** 2016-07-03

**Authors:** Christoph Heinrich Houben, Kin Wai Edwin Chan, Jennifer Wai Cheung Mou, Yuk Him Tam, Kim Hung Lee

**Affiliations:** Division of Paediatric Surgery and Paediatric Urology, Department of Surgery, Prince of Wales Hospital, Shatin, Hong Kong, China

**Keywords:** Necrotizing enterocolitis, Intestinal stricture, Primary anastomosis, Neurodevelopmental delay

## Abstract

**Objectives:** Evaluating the long-term outcome of the surgical management for intestinal strictures developing after necrotizing enterocolitis (NEC).

**Patients and methods:** This is a retrospective study of all patients with an intestinal stricture after completion of conservative management for NEC. They were treated during the eight years period from 1st January 2008 to 31st December 2015.

**Results:** During the study period 67 infants had an operation for NEC, of which 55 had emergency surgery. The remaining twelve infants (6 males) had a stricture and were included in the study group. Their median gestational age was 35 (range 27-40) weeks and the median weight was 2180 (range 770 - 3290) g. The onset of NEC was seen at a median of 2 (range 1- 47) days. The median peak C-reactive protein (CRP) level was 73.1 (range 25.2 – 232) mg/dl. Isolated strictures were seen in 9 (75%) patients. Two-third of all strictures (n=15) were located in the colon. Surgery was done at a median of 5 (range 3 - 13) weeks after diagnosing NEC. Primary anastomosis was the procedure of choice; only one needed a temporary colostomy. This cohort had no mortality during a median follow up of 6.25 (range 0.5 - 7.6) years, whilst the overall death rate for NEC was 15 (22 %). Two fifth of the group developed a neurological / sensory impairment.

**Conclusion:** One fifth of the surgical workload for NEC is related to post-NEC strictures. Most strictures are located in the colonic region. In the long-term no mortality and no surgical co-morbidities were observed.

## INTRODUCTION

Necrotizing enterocolitis (NEC) is an inflammatory disease process of the gastrointestinal tract commonly seen in the neonatal unit affecting mostly premature infants [1,2]. The mainstay medical intervention consists of abdominal decompression, bowel rest, intravenous antibiotics and parenteral nutrition; emergency surgery is required in infants with intestinal perforation or failure of medical management [3]. Intestinal strictures developing during or after the conservative management of NEC have been reported since the late 1960s and Rabinowitz has been credited with the first report of a stricture after NEC [4-6]. This study focuses on the long-term outcome of the surgical management of post-NEC strictures.

## MATERIALS AND METHODS

This retrospective study reviewed the records of all patients who had surgery for NEC between 1st January 2008 and 31st December 2015. Approved by the local ethics committee (CREC 2015.727) the demographics, presentation, results of radiological and laboratory investigations, operative findings and outcome, morbidity and mortality of the patient group were analyzed. All patients with a suspected intestinal stricture (e.g. food intolerance, recurrent abdominal distension, high gastric aspirates, radiological features of intestinal obstruction) underwent a contrast enema (CE) to confirm the stricture and its location (Fig. 1). If the CE was normal, an upper gastrointestinal study with small bowel follow through (UGI-SBFT) was added to identify the stricture (7, Fig. 1). Only patients developing an intestinal stricture as a result of conservative management for NEC were included in the study group. Patients found to have a stricture after surgery for NEC following inadequate resection of necrotic tissue during the first operation or disease progression after emergency surgery were excluded. 

**Figure:**
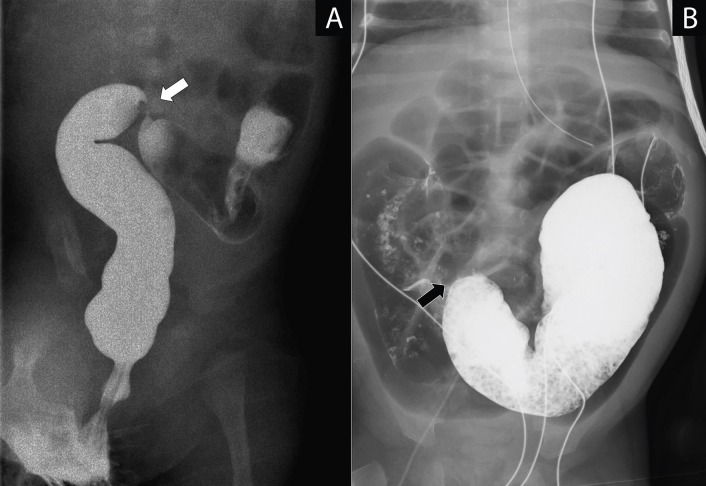
Figure 1: Panel A contrast enema with sigmoid colon stricture (white arrow) 4 weeks after onset of NEC; Panel B follow-through with distal ileum stricture (black arrow) 4 weeks after onset of NEC.

Descriptive statistical analysis were used to report the frequency and median values of patients' demographics. The results were compared with the outcome by other institutions reporting their experience with approximately 50 or more cases of NEC.

## RESULTS

During the eight years observation period a total of 67 infants underwent surgery for NEC. Fifty-five had emergency surgery for NEC, as a result of perforation or failure of medical management. Twelve (6 males) had surgery for one or more stricture(s) after completion of the conservative therapy for NEC. 

The overall mortality rate for infants undergoing surgery for NEC was 15 (22 %), but no infant died in the subgroup of post-NEC strictures as illustrated by the Kaplan-Meier curve (Fig. 2). This cohort of post-NEC stricture(s) compromised a median gestational age of 35 (range 27 - 40) weeks and a median weight of 2180 (range 770 - 3290) g; there was no evidence of intrauterine growth retardation (IUGR) within the group (Fig. 3).

**Figure:**
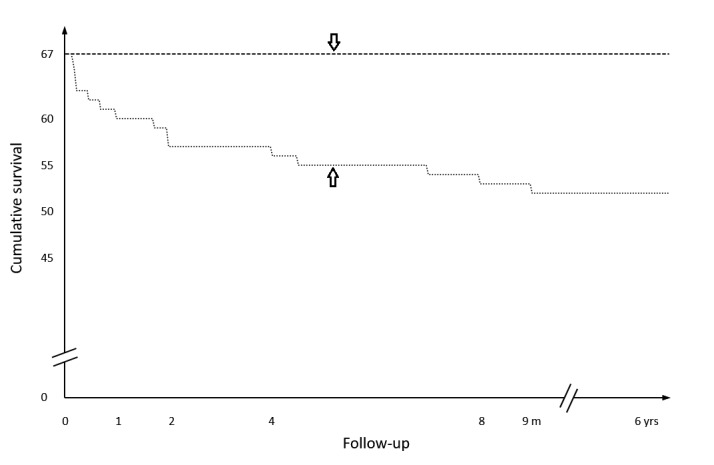
Figure 2: Kaplan-Meier survival curve showing the graph for patients undergoing emergency surgery for NEC (up-arrow) contrasted with those patients who had surgery for post-NEC stricture(s) (down-arrow).

**Figure:**
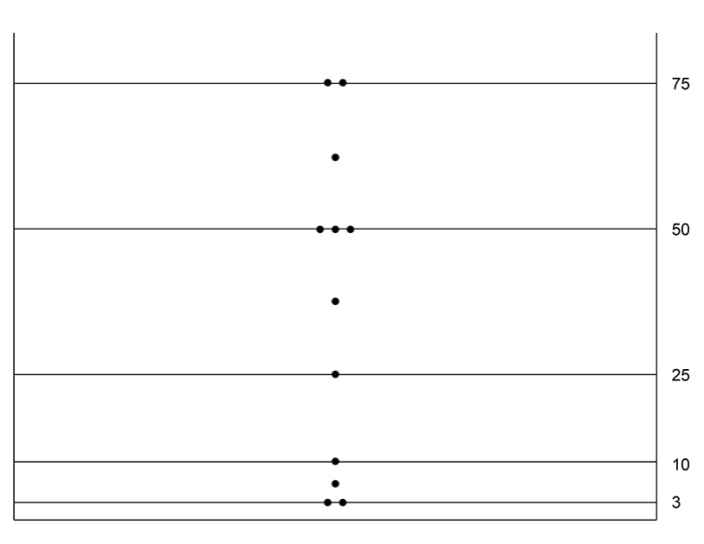
Figure 3: Percentile weight distribution of infants with NEC stricture (Note: no infants had IUGR)

NEC was diagnosed in this cohort at a median of 2 (range 1 - 47) days after birth. The median peak C-reactive protein (CRP) level during the acute phase of the NEC was 73.1 (range 25.2 - 232) mg/dl. 

A suspected intestinal stricture was confirmed by CE and if necessary clarified by an additional UGI-SBFT (Fig. 1). Isolated strictures were seen in 9 (75 %). Together with the 3 infants, who were found to have two strictures each, a total of 15 strictures were identified, of which two-thirds (10) were located in the colon (Fig. 4). Surgical resection of the stricture(s) took place at a median of 5 (range 3 - 13) weeks after diagnosing NEC. A primary anastomosis after resection of the strictured segment(s) was done in all patients except one. She required a temporary colostomy for severe size discrepancy between the transverse colon ends; the colostomy was reversed 6 weeks later. Postoperatively, two minor self-limiting wound dehiscences were recorded. 

**Figure:**
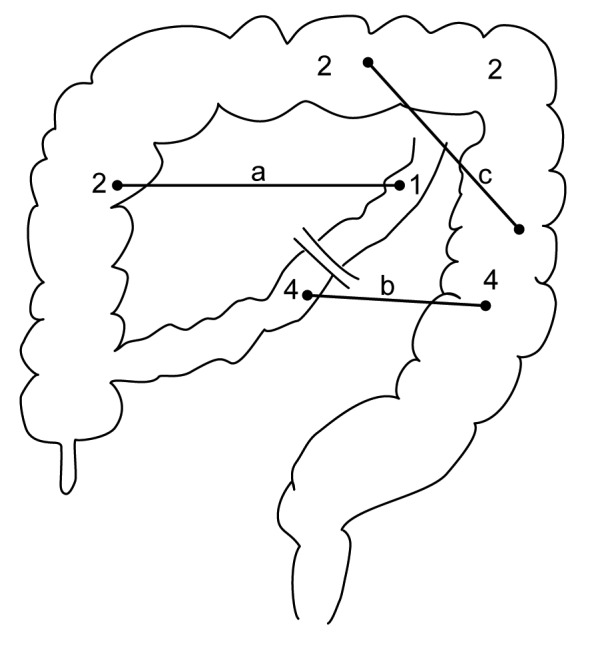
Figure 4: Graphic illustration of the position of the strictures (n=15) in 12 infants, of which 3 had two strictures each with their respective position marked by bars (a-c).

During the median follow up period of 6.25 (range 0.5 – 7.6) years this subgroup of post-NEC related strictures did not develop any further surgical complications (e.g. adhesive intestinal obstruction, anastomotic stricture). One patient's total parenteral nutrition (TPN) related cholestatic jaundice resolved. Only one infant was found to have an intra-cranial hemorrhage, but five developed conditions associated with prematurity and possibly NEC: three patients had neurodevelopmental delay and two sensory impairments, of which one required a hearing aid and one needed the correction of a divergent squint.

## DISCUSSION

This study sets out to investigate the results of the operative management of intestinal strictures, which developed during or after medical therapy for NEC; we are not concerned with intestinal strictures manifesting itself as a result of inadequate emergency surgery for NEC or disease progression after surgery. Following sporadic reports on post-NEC strictures in the 1960s a proliferation of reports concerning the management of post-NEC strictures followed in recent decades [6, 8-12].

Throughout the last four decades the colon has been by far the dominant site for stricture formation after medical therapy for NEC (Table 1). As in our cohort two-thirds had a colonic stricture either in isolation or in conjunction with another bowel narrowing (Fig. 4). A recent study by Gaudin and coworkers reported more than 80 % of colonic strictures in their analyzed group [12]. These findings justify the utilization of a contrast enema as the first line radiological intervention to identify the location and the possible extend of the stricture [7]. A contrast study should be done as soon as suspicious clinical findings (e. g. vomiting, abdominal distension, high aspirates, etc.) become apparent to avoid the development of potentially life-threatening sepsis or perforation associated with intestinal obstruction [6].

**Figure:**
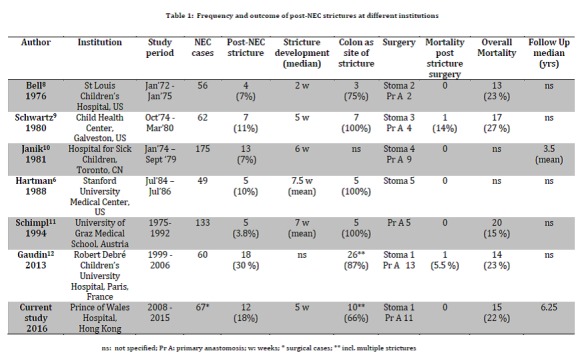
Table 1: Frequency and outcome of post-NEC strictures at different institutions

In most instances the stricture became clinically manifest at around 5 - 7 weeks after the onset of NEC (Table 1). Only Bell and coworkers intervened to resect strictures in their cohort at 2 weeks after the commencement of NEC, probably still operating during the acute phase of NEC (8).

Whilst in the 1970s and 1980s stoma formation constituted the initial step in the management of the stricture alongside primary anastomosis, we give preference to a direct anastomosis after resection of the stricture. This view is shared by Gaudin and Schimpl and their respective coworkers who prefer a primary anastomosis to a staged stoma formation except in circumstances of gross discrepancy between bowel ends [11,12].

We cannot confirm the assertion by Janik and coworkers, primary anastomosis may be hazardous before 3 months have elapsed, as histological specimen may demonstrate active inflammatory disease [10]. In our cohort all but one resection of intestinal stricture(s) were done within 3 months after the onset of the NEC; in fact the median time for surgical intervention was just 5 weeks. We did not encounter an anastomotic stricture. 

The overall mortality for NEC is around 20+ percent (Table1). However mortality in infants treated for post-NEC stricture is remarkably low (Table 1); we did not record a death at all in our cohort (Fig. 2).

The long-term results for surgical co-morbidities are also encouraging; Janik had no surgical complications after a mean observation time of 3.5 years [10]. We encountered neither an adhesive intestinal obstruction nor a recurrent stricture after a median of 6.25 years follow up.

However we observed a number of neurological and sensory sequelae in our cohort. A majority of this group were premature infants susceptible to neurodevelopmental and sensory impairment, albeit only one had an intra-cranial hemorrhage. NEC, the effects of surgery and the prolonged hospital stay for these infants may contribute to the resulting neurodevelopmental delay and sensory impairment affecting two-fifth of this group [2]. 

Post-NEC strictures accounted for 7-10 % of the overall NEC workload in the 1970s and 1980s [3-6]. Looking at our results and contemporary studies there may be a trend towards a higher rate of post-NEC strictures possibly as a result of better intensive care management for acute NEC [12]. Whilst we acknowledge surgery in NEC is frequently required at an early stage (e.g. perforation), this study shows a clear survival advantage for infants developing a post-NEC stricture.

Heida and coworkers identified no post-NEC strictures in patients with their highest CRP level less than 46 mg/ml during the acute phase of NEC [13]. We cannot confirm these findings, in fact 17 % (2/12) of our cohort had a lower maximal CRP level and still developed a stricture. It is difficult to see a marker for a generalized inflammatory process predicting localized changes as the formation of strictures.

## CONCLUSION

Intestinal strictures after conservative management for NEC constitute approximately 20 % of the surgical workload for NEC. The majority of strictures are single isolated strictures mostly in the colonic region. Surgery for post-NEC related strictures carries a low mortality risk. Our study had no long-term mortality. The long-term outcome shows a remarkably low risk for surgical co-morbidity (e.g. adhesive intestinal obstruction), but neurodevelopmental and sensory impairment affect 40 % of the cohort. 

## Footnotes

**Source of Support:** None

**Conflict of Interest:** None
